# Diagnostic accuracy of a low-cost, urine antigen, point-of-care screening assay for HIV-associated pulmonary tuberculosis before antiretroviral therapy: a descriptive study

**DOI:** 10.1016/S1473-3099(11)70251-1

**Published:** 2012-03

**Authors:** Stephen D Lawn, Andrew D Kerkhoff, Monica Vogt, Robin Wood

**Affiliations:** a The Desmond Tutu HIV Centre, Institute for Infectious Disease and Molecular Medicine, Faculty of Health Sciences, University of Cape Town, Cape Town, South Africa; b Department of Clinical Research, Faculty of Infectious and Tropical Diseases, London School of Hygiene and Tropical Medicine, London, UK; c The School of Medicine and Health Sciences, George Washington University, Washington, DC, USA

## Abstract

**Background:**

The diagnostic accuracy of sputum smear microscopy and routine chest radiology for HIV-associated tuberculosis is poor, and culture-based diagnosis is slow, expensive, and is unavailable in most resource-limited settings. We assessed the diagnostic accuracy of a urine antigen test Determine TB-LAM Ag (Determine TB-LAM; Alere, Waltham, MA, USA) for screening for HIV-associated pulmonary tuberculosis before antiretroviral therapy (ART).

**Methods:**

In this descriptive study, consecutive adults referred to a community-based ART clinic in Gugulethu township, South Africa, were all screened for tuberculosis by obtaining sputum samples for fluorescence microscopy, automated liquid culture (gold-standard test), and Xpert MTB/RIF assays (Cepheid, Sunnyvale, CA, USA) and urine samples for the Clearview TB-ELISA (TB-ELISA; Alere, Waltham, MA, USA) and Determine TB-LAM test. Patients with *Mycobacterium tuberculosis* cultured from one or more sputum samples were defined as cases of tuberculosis. The diagnostic accuracy of Determine TB-LAM used alone or combined with sputum smear microscopy was compared with that of sputum culture and the Xpert MTB/RIF assay for all patients and subgroups of patients stratified by CD4 cell count.

**Findings:**

Patients were recruited between March 12, 2010, and April 20, 2011. Of 602 patients enrolled, 542 were able to provide one or more sputum samples, and 94 had culture-positive tuberculosis (prevalence 17·4%, 95% CI 14·2–20·8). Complete results from all tests were available for 516 patients (median CD4 count, 169·5 cells per μL; IQR 100–233), including 85 culture-positive tuberculosis, 24 of whom (28·2%, 95% CI 19·0–39·0) had sputum smear-positive disease. Determine TB-LAM test strips provided results within 30 min. Agreement was very high between two independent readers of the test strips (κ=0·97) and between the test strips and TB-ELISA (κ=0·84). Determine TB-LAM had highest sensitivity at low CD4 cell counts: 66·7% (95% CI 41·0–86·7) at <50 cells per μL, 51·7% (32·5–70·6) at <100 cells per μL, and 39·0% (26·5–52·6) at <200 cells per μL; specificity was greater than 98% for all strata. When combined with smear microscopy (either test positive), sensitivity was 72·2% (95% CI 46·5–90·3) at CD4 counts less than 50 cells per μL, 65·5% (45·7–82·1) at less than 100 cells per μL, and 52·5% (39·1–65·7) at less than 200 cells per μL, which did not differ statistically from the sensitivities obtained by testing a single sputum sample with the Xpert MTB/RIF assay.

**Interpretation:**

Determine TB-LAM is a simple, low-cost, alternative to existing diagnostic assays for tuberculosis screening in HIV-infected patients with very low CD4 cell counts and provides important incremental yield when combined with sputum smear microscopy.

**Funding:**

Wellcome Trust.

## Introduction

Tuberculosis is a leading cause of morbidity and mortality in patients accessing antiretroviral treatment (ART) programmes in sub-Saharan Africa and much of this disease remains unidentified.^1^, ^2^, ^3^, ^4^ The high burden of disease in these often overcrowded clinical services also presents a substantial risk of nosocomial disease transmission.^5^ Intensified case finding is therefore a key intervention in these settings,^6^, ^7^ enabling early diagnosis and rapid initiation of treatment. However, case finding among patients with advanced HIV-associated immunodeficiency still presents a major challenge since the diagnostic accuracy of the most widely used diagnostic tools (sputum-smear microscopy and chest radiology) is poor and culture-based diagnosis is slow, expensive, and is mostly unavailable in resource-limited settings due to the complex technical infrastructure required.^8^

We previously reported that the Xpert MTB/RIF assay (Cepheid, Sunnyvale, CA, USA) substantially increased case finding compared with sputum-smear microscopy when used to routinely screen for tuberculosis in an ART service in Cape Town, South Africa, allowing rapid diagnosis of about two-thirds of culture-confirmed cases.^9^ This assay represents a substantial technological advance in tuberculosis diagnostics that can be used in the clinical environment by operators with little technical training.^10^ However, the assay has drawbacks: it is expensive, requires sophisticated hardware with annual servicing, must be linked to a personal computer, and requires an electricity supply. The assay has been endorsed by WHO^11^ and yet implementation and scale-up is likely to be hindered by the high cost, which might be judged prohibitive in many poor countries.

The need for a simple, low-cost, point-of-care assay for tuberculosis is great.^12^ We and others have described the usefulness of a simple, commercially available ELISA that detects lipoarabinomannan (LAM) in urine as a diagnostic test for tuberculosis.^13^, ^14^, ^15^, ^16^, ^17^, ^18^, ^19^, ^20^, ^21^ LAM is a 19 kDa lipopolysaccharide major cell-wall component of *Mycobacterium tuberculosis*^22^ that can be detected in urine of patients with pulmonary and extrapulmonary disease.^20^, ^21^ Although the sensitivity of this assay (about 10–20%) in non-HIV-infected individuals has been disappointing,^20^ moderate sensitivity and high specificity have been reported in HIV-infected individuals with advanced immunodeficiency,^13^, ^14^ the very patients in whom tuberculosis is especially difficult to diagnose.

In this study, we assess the diagnostic accuracy of a new point-of-care lateral-flow urine test for lipoarabinomannan (LAM; Determine TB-LAM Ag [Determine TB-LAM] Alere, Waltham, MA, USA) and the most recent version of the commercially available Clearview TB ELISA (TB-ELISA; Alere, Waltham, MA, USA) for screening a cohort of patients for tuberculosis before starting ART in South Africa.

## Methods

### Patients

The ART service in Gugulethu township, Cape Town, South Africa, has previously been described in detail as has the major burden of tuberculosis in this service.^2^, ^23^, ^24^ We did a descriptive study of consecutive HIV-infected patients newly referred to the clinic in Gugulethu township for ART. Patients were prospectively recruited and investigated at their first visit to the clinic. Patients were eligible for the study if they were older than 18 years of age, ART-naive, and had no current tuberculosis diagnosis. All participants provided written informed consent and the study was approved by the research ethics committees of the University of Cape Town, South Africa, and the London School of Hygiene & Tropical Medicine, UK. The study protocol was prespecified and was reported in conformity with the STAndards for the Reporting of Diagnostic accuracy studies.

### Procedures

Demographic details were recorded and a standardised symptom-screening questionnaire was completed. Two sputum samples were requested from each patient: a spot specimen was obtained first followed by a second that was induced with nebulised 3% hypertonic saline. If necessary, both specimens were induced. Urine samples were also collected in sterile containers and stored at –20°C within 3 h of collection. Blood CD4 cell counts and plasma viral load were measured for all patients via the routine laboratory services. Chest radiographs were obtained for all patients except pregnant women and were assessed by an experienced investigator (SDL) certified in the use of the chest-radiograph reading and recording system.^25^, ^26^ Radiographs were scored according to the presence of any radiographic abnormality consistent with (but not necessarily suggestive of) a diagnosis of tuberculosis.

Sputum specimens were processed with standardised protocols and quality assurance procedures by a centralised accredited laboratory. After decontamination with N-acetyl-L-cysteine and sodium hydroxide, centrifuged sputum deposits underwent microscopy, and after resuspension in phosphate buffer, equal volumes were tested by culture and the Xpert MTB/RIF assay. Smears stained with auramine O fluorescent stain were examined with fluorescence microscopy. All sample smears graded as scanty, 1+, 2+, and 3+ were defined as smear-positive. Mycobacterial growth indicator tubes (MGIT; Becton Dickinson, Sparks, MD, USA) were inoculated and incubated for up to 6 weeks. Culture isolates positive for acid-fast bacilli were identified as *M tuberculosis* complex with the MTBDRplus assay (Hain Lifesciences, Nehren, Germany). Acid-fast bacilli that tested negative for *M tuberculosis* with the MTBDRplus assay were assumed to be non-tuberculous mycobacteria but were not speciated further. Xpert MTB/RIF and MTBDRplus assays were done according to the manufacturer's instructions. Results of all tests were read by technologists who were masked to the outcomes of the other assays.

Frozen urine samples were defrosted and analysed for the presence of LAM with the commercially available Clearview TB ELISA with strict adherence to the manufacturer's instructions. Urine samples were prepared by heating to 95–100°C for 30 min and, after cooling, were centrifuged at 10 000 rpm for 15 min. Supernatants were analysed in duplicate together with positive and negative controls. An optical density reading of 0·1 or higher above the negative control was scored as positive in accordance with the manufacturer's instructions.

Urine samples were also tested in the laboratory with the Determine TB-LAM test (a single manufacturing lot #101102 was used). Samples were defrosted to ambient temperature. For each sample, 60 μL was applied to the sample pad at the bottom of the test strip. Between 25 min and 35 min later, the test was read under ambient laboratory lighting conditions by two investigators (SDL and MV), who compared the test strips with a standardised reference card to facilitate reading. Each reader independently recorded the results, taking note of both the positive control bar and the sample test result. After comparison of the results, any discrepancies were discussed and the test strip reviewed within the 25–35 min time frame to reach consensus.

### Statistical analysis

The study population was characterised with simple descriptive statistics and patients with and without tuberculosis were compared with the Wilcoxon rank-sum test, *t*-test, χ^2^-square test, and Fisher's exact test accordingly. Patients with *M tuberculosis* cultured from one or more sputum samples were defined as cases of tuberculosis. Results from the TB-ELISA and the Determine TB-LAM strip tests were compared and the proportionate agreement and κ statistics were calculated. The sensitivity, specificity, and predictive values of the different tuberculosis diagnostic assays were calculated with 95% CIs. These parameters were also calculated for subgroups of patients stratified by CD4 cell count, WHO stage, symptoms, and radiological abnormalities. Venn diagrams were plotted to compare the diagnostic yields of the different tests. All statistical tests are two-sided at α=0·05.

### Role of the funding source

The sponsor of the study had no role in study design, data collection, data analysis, data interpretation, or writing of the report. The corresponding author had full access to all the data in the study and had final responsibility for the decision to submit for publication.

## Results

Patients were recruited between March 12, 2010, and April 20, 2011, some of whom have previously been described.^9^ Of 604 consecutive eligible patients who were invited, 602 agreed to participate. Urine samples were obtained from 595 (99%) patients. Sputum samples were obtained from 542 (90%) patients, and *M tuberculosis* was cultured from one or both samples from 94 patients. Thus, the prevalence of pulmonary tuberculosis in patients from whom sputum could be obtained was 17·3% (95% CI 14·2–20·8). The prevalence of tuberculosis in patients with CD4 counts of less than 100 cells per μL was 26·4% (95% CI 19·3–34·5), with less than 200 cells per μL was 19·5% (95% CI 15·4–24·1), and 200 or more cells per μL was 13·3% (95% CI 8·9–18·9).

**Figure 1:**
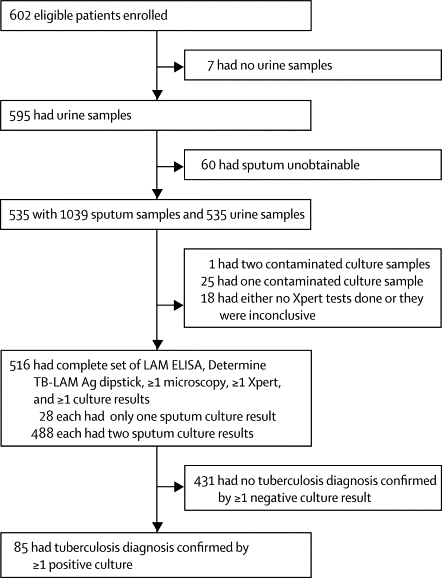
Study profile

Complete sets of smear microscopy, culture, and Xpert MTB/RIF tests (from one or both sputum samples) and urine LAM ELISA and urine Determine TB-LAM results were available from a total of 516 patients (figure 1). All subsequent analyses were based on these 516 patients, of whom 85 had culture-positive tuberculosis and 431 had sputum cultures negative for *M tuberculosis*. Additionally, non-tuberculous mycobacteria were cultured from samples of eight patients with CD4 counts of 0–99 cells per μL (four), 100–199 cells per μL (three), and 200 or more cells per μL (one). All eight also tested negative for *M tuberculosis* with the Xpert MTB/RIF assay.

The study participants were mostly young adults, of whom most were female (table 1). The median CD4 cell count was 169·5 cells per μL (IQR 100–233), about two-thirds of patients had a CD4 cell count of less than 200 cells per μL, and about a third of patients were assessed as having WHO stage 3 or stage 4 (AIDS) disease before tuberculosis screening (table 1). Patients diagnosed with tuberculosis had lower CD4 cell counts and were more likely to have advanced WHO stage of disease than were those with higher CD4 cell counts (table 1). A positive WHO symptom screen was recorded in over two-thirds of all study participants and most patients with tuberculosis. Radiological abnormalities consistent with pulmonary tuberculosis were seen in just three-quarters of patients with tuberculosis but were also seen in about two-fifths of patients without tuberculosis.

**Table 1:** Patient characteristics

		**All patients (n=516)**	**Tuberculosis diagnosed (n=85)**	**No tuberculosis diagnosed (n=431)**	**p value**
Age		34·1 (28·6–41·3)	33·4 (28·7–40·7)	34·1 (28·5–41·3)	0·806
Female		331 (64%)	52 (61%)	279 (65%)	0·532
BMI		23·5 (20·9–27·1)	21·2 (19·3–25·9)	23·9 (21·1–27·2)	0·0001
CD4 counts (cells per μL)*		169·5 (100–233)	139 (65·5–205)	172 (108–237)	0·006
	<50	64 (12%)	18 (21%)	46 (11%)	0·064
	50–99	64 (12%)	11 (13%)	53 (12%)	..
	100–149	96 (19%)	17 (20%)	79 (18%)	..
	150–199	101 (20%)	13 (15%)	88 (20%)	..
	≥200	189 (37%)	25 (30%)	164 (38%)	..
Baseline viral load (log copies per mL)†		4·6 (4·1–5·0)	4·8 (4·4–5·3)	4·5 (4·0–5·0)	0·0001
WHO stage at enrolment					
	1 or 2	346 (67%)	47 (55%)	299 (69%)	0·012
	3 or 4	170 (33%)	38 (45%)	132 (31%)	..
Positive WHO symptom screen		356 (69%)	70 (82%)	286 (66%)	0·004
Previous history of tuberculosis		140 (27%)	19 (22%)	121 (28%)	0·278
Current cough ≥2 weeks		104 (20%)	21 (25%)	83 (19%)	0·252
Radiological abnormality consistent with tuberculosis‡		235 (50%)	62 (76%)	173 (45%)	<0·0001

Data are median (IQR) or number (%). BMI=body mass index.

* CD4 cell counts available for 84 patients with tuberculosis and 430 patients without tuberculosis.

† Viral loads were available for 513 patients.

‡ Chest radiographs were available for 470 patients.

**Figure 2:**
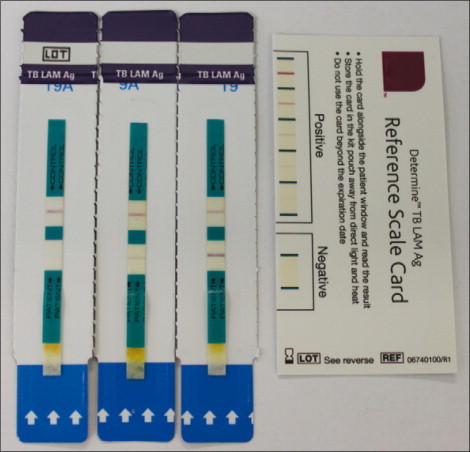
Determine TB-LAM test strips from three patients in this study together with the reference reading card Positive control (upper) bands are seen in all three strips whereas positive test (lower) bands are only seen in the middle and right hand strips and the left strip is negative.

We compared the two independently observed readings of the Determine TB-LAM urine test strips for 516 patients. Examples of the developed test strips are shown for three patients in figure 2. All test strips had a positive control bar and were therefore readable. The proportionate agreement between the readers was 514 (99·6%, 95% CI 98·6–100) of 516 (κ 0·97, 95% CI, 0·88–0·99). The two discrepancies were resolved by consensus with the two observers remaining masked to the other laboratory results.

Positive Determine TB-LAM results were noted in 24 samples from patients with culture-positive tuberculosis with a median optical density above background of 0·681 (IQR 0·164–2·431; range 0·102–3·291). Among patients with culture-negative sputum samples (431), six had positive test-strip results whereas the remaining 425 samples were negative. The proportionate agreement between the TB-ELISA and urine test strips was 507 (98·3%; 95% CI 96·7–99·2) of 516 (κ statistic 0·84, 95% CI 0·72–0·92), suggesting very good agreement. Of the nine discordant results, three were for patients with culture-positive tuberculosis (ELISA-negative *vs* Determine-positive [one] and ELISA-negative *vs* Determine-positive [two]). The six other discordant results were from those who were sputum culture-negative (ELISA-positive *vs* Determine-negative [four] and ELISA-negative *vs* Determine-positive [two]). Thus, the Determine TB-LAM assay had marginally higher sensitivity and specificity than did the TB-ELISA assay.

Compared with the gold standard of automated liquid culture, the overall sensitivity of sputum-smear microscopy was low, as were the sensitivities of both the TB-ELISA and Determine TB-LAM strip test (table 2). The specificities of the three assays, however, each exceeded 98% (table 3). Overall sensitivity was modestly increased when smear microscopy and the Determine TB-LAM strip test were used in combination (either test positive), while retaining high specificity (both tests negative).

**Table 2:** Sensitivity of the different diagnostic assays for all patients with tuberculosis and for those stratified by CD4 cell count

	**Sputum AFB**		**TB ELISA**		**Determine TB-LAM**		**Determine TB-LAM and sputum AFB**		**Xpert MTB/RIF (1 sample)**		**Determine TB-LAM and Xpert MTB/RIF**	
	Positive	Sensitivity	Positive	Sensitivity	Positive	Sensitivity	Positive	Sensitivity	Positive	Sensitivity	Positive	Sensitivity
All (n=85)	24	28·2% (19·0–39·0)	23	27·1% (18·0–37·8)	24	28·2% (19·0–39·0)	37	43·5% (32·8–54·7)	49	57·6% (46·4–68·3)	52	61·2% (50·0–71·6)
<50 cells per μL (n=18)	6	33·3% (13·3–59·0)	11	61·1% (35·7–82·7)	12	66·7% (41·0–86·7)	13	72·2% (46·5–90·3)	13	72·2% (46·5–90·3)	15	83·3% (58·6–96·4)
<100 cells per μL (n=29)	10	34·5% (17·9–54·3)	14	48·3% (29·4–67·5)	15	51·7% (32·5–70·6)	19	65·5% (45·7–82·1)	22	75·9% (56·5–89·7)	24	82·8% (64·2–94·2)
<150 cells per μL (n=46)	16	34·8% (21·4–50·2)	20	43·5% (28·9–58·9)	21	45·7% (30·9–61·0)	27	58·7% (43·2–73·0)	33	71·7% (56·5–84·0)	35	76·1% (61·2–87·4)
<200 cells per μL (n=59)	18	30·5% (19·2–43·9)	21	35·6% (23·6–49·1)	23	39·0% (26·5–52·6)	31	52·5% (39·1–65·7)	37	62·7% (49·1–75·0)	40	67·8% (54·4–79·4)
≥200 cells per μL (n=25)	6	24·0% (9·4–45·1)	2	8·0% (1·0–26·0)	1	4·0% (0·1–20·4)	6	24·0% (9·4–45·1)	11	44·0% (24·4–65·1)	11	44·0% (24·4–65·1)

84 patients stratified by CD4 cell count. Data are number and sensitivity (95% CI). AFB=acid-fast bacilli. LAM=lipoarabinomannan. MTB/RIF=*Mycobacterium tuberculosis*/rifampicin.

**Table 3:** Specificity of the different diagnostic assays for all patients with tuberculosis whose cultures were negative

	**Sputum AFB**		**ELISA**		**Determine TB-LAM**		**Determine TB-LAM and sputum AFB**		**Xpert MTB/RIF (1 sample)**		**Determine TB-LAM and Xpert MTB/RIF**	
	Negative	Specificity	Negative	Specificity	Negative	Specificity	Negative	Specificity	Negative	Specificity	Negative	Specificity
All (n=431)	430	99·8% (98·7–100)	423	98·1% (96·4–99·2)	425	98·6% (97·0–99·5)	424	98·4% (96·7–99·3)	427	99·1% (97·6–99·7)	421	97·7% (95·8–98·9)

Data are number and sensitivity (95% CI). AFB=acid-fast bacilli. TB=tuberculosis. LAM=lipoarabinomannan. MTB/RIF=*Mycobacterium tuberculosis*/rifampicin.

The potential usefulness of the Determine TB-LAM assay was evident in patients with advanced immunodeficiency when data were stratified by CD4 cell count (table 2). The assay correctly identified more than two-thirds of tuberculosis cases with CD4 counts less than 50 cells per μL and about half of cases with counts less than 100 cells per μL. When use was expanded to also include patients with higher CD4 cell counts, the sensitivity gradually declined and was very low above a threshold of 200 cells per μL (table 2). However, when Determine TB-LAM strip tests were used in combination with smear microscopy (either test positive), an important additive effect occurred: sensitivities for patients with CD4 counts less than 50, less than 100, and less than 200 cells per μL were increased. The specificity (both tests negative) across all strata was 98% or higher.

We also calculated the sensitivity of Determine TB-LAM strip tests for culture-positive patients with WHO stage 3 or 4 disease (46·0%; 95% CI 29·5–63·1), a positive WHO symptom screen (31·4%; 95% CI, 20·9–43·6), and for those with an abnormal chest radiograph (27·9%; 95% CI 17·1–40·8). When Determine TB-LAM test strips and smear-microscopy results were combined (either test positive), the sensitivities for these three groups were 59·5% (stage 3 or 4), 48·6% (positive symptom screen), and 45·9% (abnormal chest radiograph). In all these subgroup analyses, the specificity of the tests for culture-negative patients was greater than 98% for each subgroup with the Determine TB-LAM test alone or combined with smear microscopy (table 3).

Among six patients with negative sputum cultures for *M tuberculosis* but whose urine samples tested positive with Determine TB-LAM, all had sputum samples that tested negative with the Xpert MTB/RIF assay. Extrapulmonary tuberculosis was not clinically suspected in any of the six patients at the time of screening, but non-tuberculous mycobacteria were cultured from sputum samples from two of them. Only two of these patients had a CD4 cell count less than 150 cells per μL or WHO stage 3 or 4 disease—the subgroups in which the assay has the greatest usefulness.

To further assess the effectiveness of Determine TB-LAM in different patient subgroups with differing prevalence of tuberculosis, we calculated the positive predictive and negative predictive values when using the test alone or combined with smear microscopy (table 4). When Determine TB-LAM was applied to all patients irrespective of their characteristics, the positive predictive value was lower than if selectively applied to patients with CD4 cell counts in the range 0–150 cells per μL or to patients with WHO stage 3 or 4 disease. In all subgroups, the negative predictive value varied substantially (table 4).

**Table 4:** Positive and negative predictive values of Determine TB-LAM or Determine TB-LAM and smear microscopy combined when applied to different patient subgroups with differing tuberculosis prevalence

		**TB prevalence**	**Determine TB-LAM**		Determine TB-LAM and sputum AFB*	
			Positive predictive value	Negative predictive value	Positive predictive value	Negative predictive value
All patients (n=516)		16·5% (13·4–20·0)	80·0% (61·4–92·3)	87·4% (84·2–90·3)	84·1% (69·9–93·4)	89·8% (86·7–92·4)
CD4 cell count (cells per μL)†						
	<50	28·1% (17·6–40·8)	92·3% (64·0–99·8)	90·2% (78·6–96·7)	92·9% (66·1–99·8)	90·0% (78·2–96·7)
	<100	22·7% (15·7–30·9)	93·8% (69·8–99·8)	87·5% (79·9–93·0)	95·0% (75·1–99·9)	90·7% (83·6–95·5)
	<150	20·5% (15·4–26·4)	91·3% (72·0–98·9)	87·6% (82·2–91·8)	93·1% (77·2–99·2)	90·3% (85·2–94·0)
	<200	18·2% (14·1–22·8)	88·5% (69·8–97·6)	88·0% (83·7–91·4)	88·6% (73·3–96·8)	90·3% (86·3–93·5)
	≥200	13·2% (8·7–18·9)	66·7% (9·4–99·2)	87·1% (81·4–91·6)	75·0% (34·9–96·8)	89·5% (84·1–93·6)
WHO stage						
	Stage 1 or 2	13·6% (10·2–17·7)	63·6% (30·8–89·1)	88·0% (84·1–91·3)	78·9% (54·4–93·9)	90·2% (86·4–93·2)
	Stage 3 or 4	22·4% (16·3–29·4)	94·4% (72·7–99·9)	86·8% (80·3–91·7)	91·7% (73·0–99·0)	89·7% (83·5–94·1)
WHO symptom screen						
	Positive	19·7% (15·7–24·2)	84·6% (65·1–95·6)	85·4% (81·1–89·0)	87·2% (72·6–95·7)	88·6% (84·6–91·9)
	Negative	9·4% (5·3–15·0)	66·7% (9·4–99·2)	92·3% (86·9–96·0)	75·0% (19·4–99·4)	93·5% (88·4–96·8)
Any chest-radiograph abnormality‡		26·4% (20·9–32·5)	85·0% (62·1–96·8)	79·4% (73·4–84·6)	90·3% (74·2–98·0)	83·7% (77·9–88·5)

Data are % (95% CI) or positive or negative predictive value (95% CI). AFB=acid-fast bacilli. TB=tuberculosis. LAM=lipoarabinomannan.

* When assessing the predictive values of combined results of Determine TB-LAM and smear microscopy, the positive predictive value was based on either test being positive, and the negative predictive value was based on both tests being negative.

† 514 patients.

‡ 470 patients.

When the results of Determine TB-LAM were combined with those of smear microscopy, the positive predictive value when testing unselected patients was lower than when applied to patients with CD4 cell counts in the range 0–150 cells per μL and for those with WHO stage 3 or 4 disease and radiographic abnormalities (table 4).

**Figure 3:**
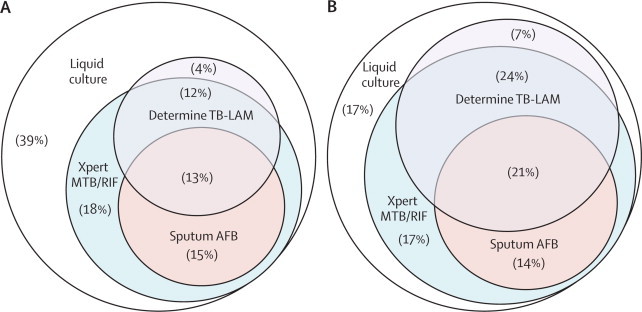
Venn diagrams showing the proportions of patients diagnosed by sputum culture, Xpert MTB/RIF (1 sample), sputum-smear microscopy (sputum acid-fast bacilli), and Determine TB-LAM Mutually exclusive proportions of patients shown in each of the compartments. Proportions of all patients with tuberculosis (A; n=85). Proportions of patients with CD4 cell counts less than 100 cells per μL (B; n=29). AFB=acid-fast bacilli.

We next compared the diagnostic effectiveness of the Determine TB-LAM test strips and smear microscopy with that of the Xpert MTB/RIF assay. When screening all patients irrespective of CD4 cell count, the sensitivity and specificity of the Xpert MTB/RIF when testing a single sputum sample was greater than the Determine TB-LAM test strips and microscopy (Table 2, Table 3, figure 3). However, when screening patients with advanced immunodeficiency, the combined sensitivity of Determine TB-LAM test strips and smear microscopy combined (either test positive) did not differ significantly from that of the Xpert MTB/RIF assay for patients with CD4 cell counts less than 50 cells per μL or less than 100 cells per μL (figure 3, table 2). A small increment in sensitivity was also seen when results from both Xpert MTB/RIF and Determine TB-LAM were combined (figure 3, table 2).

Of 516 patients included in the analysis, 478 had data available for Xpert MTB/RIF tests on two sputum samples. In this group of patients, the sensitivity of Determine TB-LAM and smear microscopy combined was 41·0% (95% CI 30·0–52·7) versus 66·7% (55·1–76·9) for Xpert MTB/RIF (two samples; p=0·002). Among 115 patients with CD4 counts less than 100 cells per μL, the sensitivities were 62·5% (95% CI 40·6–81·2) for Determine TB-LAM and smear microscopy combined and 79·2% (57·8–92·9) for Xpert MTB/RIF with two samples (p=0·34). Moreover, in this subset, the sensitivity of Determine TB-LAM and Xpert MTB/RIF (one sample) was identical to that of Xpert MTB/RIF (two samples) both 79·2% (95% CI 57·8–92·9). In all these subanalyses, specificity was 98% or higher.

## Discussion

The Determine TB-LAM lateral-flow urine antigen test was simple to use with extremely high inter-observer agreement between two independent readers. Compared with a diagnostic gold standard of liquid culture of sputum samples, the assay was useful for screening for pulmonary tuberculosis in patients with advanced HIV-associated immunodeficiency in this very high burden setting (panel). Specificity of the assay was high in all analyses (table 3), including those relating to a range of subgroups. Sensitivity was strongly associated with patients' CD4 cell count, with the highest sensitivity recorded for those less than 50 cells per μL—the very patients for whom rapid diagnosis is most urgently needed. An important additive effect occurred when results were combined with sputum smear microscopy (table 2). These combined results in patients with very low CD4 counts were similar to the sensitivity obtained when testing one sputum sample with the Xpert MTB/RIF assay.

Panel **Research in context**

**Systematic review**

We searched PubMed for articles published between Jan 1, 1990, and August 1, 2011, with the key words “tuberculosis”, “diagnosis”, and “lipoarabinomannan”. We included original research studies that provided data about the diagnostic accuracy of commercially available diagnostic assays that detect lipoarabinomannan (LAM) in urine for the diagnosis of tuberculosis in HIV-infected patients.

**Interpretation**

Our study is the first to assess the effectiveness of a simple lateral-flow point-of-care assay for detection of urinary LAM for the diagnosis of tuberculosis during routine screening of HIV-infected patients. The point-of-care version of the assay had diagnostic accuracy consistent with that recorded when urine samples from HIV-infected individuals with advanced immunodeficiency were tested with a laboratory-based ELISA, but it was much easier to use, providing results within 25 min without need for equipment or laboratory training. The assay is likely to be useful as a rapid point-of-care tuberculosis diagnostic method in this group of patients.

The prevalence of tuberculosis in this and other ART cohorts in southern Africa^3^, ^9^, ^13^ is very high. About a third of patients referred to the ART service in Gugulethu township and other ART clinics in South Africa had a pre-existing tuberculosis diagnosis and so were ineligible for inclusion.^27^ However, such patients had a very similar proportion of smear-positive disease to that detected in the patients we diagnosed (32% *vs* 28%), suggesting little selection bias with regard to the type of tuberculosis.^28^ We screened all patients irrespective of the presence or absence of symptoms and reported a high prevalence of sputum culture-positive pulmonary tuberculosis in patients with CD4 counts less than 200 cells per μL. In view of this finding and the limited sensitivity of symptom screening tools, microbiological testing for tuberculosis has been suggested for all HIV-infected patients in such high-burden settings before starting ART, regardless of the presence or absence of symptoms.^3^, ^9^, ^13^ To date, however, the high cost of culture, limited laboratory capacity, the challenges of safely obtaining sputum samples in the clinic environment, and the administrative challenges associated with linking results from centralised laboratories with patients' records in over-stretched clinical services have made application of such a policy difficult. Moreover, availability of routine culture-based diagnosis of tuberculosis is extremely poor in sub-Saharan Africa and new alternative diagnostic strategies are clearly needed.

Studies done in countries with high tuberculosis burden have shown that diagnostic tests that detect urine LAM have the greatest potential usefulness in HIV-infected patients with advanced immunodeficiency such as those admitted to hospital and those enrolling to start ART.^13^, ^14^ However, this test is in the format of a 96-well ELISA assay and requires that urine samples undergo initial processing with incubation at 95–100°C for 30 min followed by high-speed centrifugation. Thus, the assay can only be done in a laboratory setting and with batched sample processing. The Determine TB-LAM test strips therefore provide a substantial advance. The test strips provided sensitivity and specificity that was comparable with the laboratory-based tuberculosis ELISA and results were also extremely consistent with those obtained in previous studies.^13^, ^14^ No prior sample processing is required and the simple lateral-flow format of the assay can be very readily used on a per-patient basis at the point-of-care by health-care personnel with no laboratory training. Results are read after 25 min incubation and so can be available during a single clinic visit. The assay is applied to urine samples, which are easy to obtain, have low biohazard risk, and do not generate infectious aerosols as is the problem with sputum expectoration. Test strips do not need refrigerated storage and the assay does not generate large amounts of biohazardous waste.

Less than a third of tuberculosis cases could be diagnosed by sputum-smear microscopy. However, combination of results from Determine TB-LAM tests strips and sputum-smear microscopy improved diagnosis of tuberculosis (table 2). Determine TB-LAM could very easily be added into the diagnostic algorithm in settings where smear microscopy remains the only microbiological test available and would provide important incremental sensitivity.

The positive predictive value of Determine TB-LAM when used alone or in combination with sputum-smear microscopy was high for patients with CD4 counts less than 150 cells per μL and in those with WHO stage 3 or 4 disease but was low when the test was applied to patients with less advanced immunodeficiency. The negative predictive value of the assay was not sufficiently high to rule out a diagnosis of tuberculosis. Thus, the assay should be restricted for use as a test for tuberculosis in patients with advanced immunodeficiency. These are the patients in whom tuberculosis diagnosis is so challenging such that, in the absence of suitable diagnostic assays, empirical treatment has been suggested as a strategy to reduce the high mortality of patients with very low CD4 cell counts in settings with the highest disease burden.^29^ However, with the development of this simple point-of-care assay, such a strategy might prove unnecessary. Studies of the use of this assay and its effects on clinical outcomes in such patient groups are now needed.

The effectiveness of this moderate sensitivity assay is dependent on high specificity. Five studies of various versions of the TB LAM-ELISA in South Africa and Tanzania have all reported high specificity (96–100%).^13^, ^14^, ^15^, ^16^, ^19^ However, two other studies from sub-Saharan Africa have shown much lower specificities.^17^, ^18^ The reason for this heterogeneity is unknown, but it could be related to study design, setting, or sensitivity of the laboratory gold standard (mycobacterial culture) for tuberculosis diagnosis. No study has reported cross-reactivity with non-tuberculous mycobacteria as a cause for lowered specificity although we reported that such bacteria were associated with two false-positives. Further studies are urgently needed to assess diagnostic accuracy in other settings and to establish whether the assay can be used in isolation for reliable rapid tuberculosis diagnosis or whether confirmatory tests are subsequently needed.

In this clinical setting, we have previously reported that the Xpert MTB/RIF assay had sensitivities of 58% when testing one sputum sample and 78% when testing two samples. However, even the cost of one cartridge (US$18 at the time of the study) would represent the annual total health spending per head in many poor countries.^30^ The Determine TB-LAM assay is currently marketed at about $3·50 per test strip and thus the use of this simple test in combination with smear microscopy is a low-cost alternative that has similar sensitivity to a single Xpert MTB/RIF test for patients with CD4 counts less than 200 cells per μL. In settings in which the Xpert MTB/RIF assay is implemented, the cost of two Xpert MTB/RIF tests to gain incremental sensitivity might be prohibitively expensive. However, a single Xpert MTB/RIF test might alternatively be used in combination with a Determine TB-LAM test because this combination had identical sensitivity to two Xpert MTB/RIF tests in those with CD4 counts less than 100 cells per μL.

The strengths of this study include the assessment of Determine TB-LAM in a well characterised community-based ART service in the public sector, which is similar to other services in southern Africa. The assay was compared with a range of other tests including a rigorous gold standard for pulmonary tuberculosis (ie, automated liquid culture of sputum done in an accredited laboratory with rigorous quality assurance procedures). Sputum samples could not be obtained from 10% of enrolled patients, and in the absence of the diagnostic gold standard these patients were excluded from the analysis. The results therefore cannot be generalised to apply to this subgroup.

Although tuberculosis diagnoses can be established from sputum culture for most patients with advanced HIV-associated immunodeficiency,^31^ a few require extrapulmonary samples. Since no additional tests were done for extrapulmonary tuberculosis, the specificity of the urine LAM assays could have been underestimated or the sensitivity overestimated. Patients were carefully screened at a single timepoint but follow-up of clinical outcomes was not included in the definition of tuberculosis cases. We did not include these outcomes because the clinical presentation of tuberculosis is so non-specific in this population and the ongoing incidence of the disease is so high that the distinction between prevalent and incident tuberculosis can only be reliably made with specific microbiological endpoints in samples obtained at the time of initial screening.^32^

Although LAM is a stable lipopolysaccharide and sample freezing is acceptable before testing by the tuberculosis ELISA, the effect of freezing and thawing of the urine samples before testing with Determine TB-LAM was not assessed. Determine TB-LAM testing was not done at the actual point-of-care and results were not used to direct management of patients. Thus, the effect of the assay on outcomes is not known. Further prospective assessments of this new assay at the point-of-care are needed in different settings as is assessment of cost-effectiveness.

Determine TB-LAM test was very easy to use and is readily applicable as a point-of-care diagnostic test for HIV-associated tuberculosis when screening patients with advanced immunodeficiency. This test is an important low-cost alternative diagnostic test for this group of patients for use in resource-limited settings.
